# Assessing Radiation Hardness of Silicon Photonic Sensors

**DOI:** 10.1038/s41598-018-31286-9

**Published:** 2018-08-29

**Authors:** Zeeshan Ahmed, Lonnie T. Cumberland, Nikolai N. Klimov, Ileana M. Pazos, Ronald E. Tosh, Ryan Fitzgerald

**Affiliations:** Physical Measurement Laboratory, National Institute of Standards and Technology, Gaithersburg, MD USA

## Abstract

In recent years, silicon photonic platforms have undergone rapid maturation enabling not only optical communication but complex scientific experiments ranging from sensors applications to fundamental physics investigations. There is considerable interest in deploying photonics-based communication and science instruments in harsh environments such as outer space, where radiation damage is a significant concern. In this study, we have examined the impact of cobalt-60 γ-ray radiation up to 1 megagray (MGy) absorbed dose on silicon photonic devices. We do not find any systematic impact of radiation on passivated devices, indicating the durability of passivated silicon devices under harsh conditions.

## Introduction

The last three decades have witnessed an exponential growth in photonics, driven in part by improvements in micro-electronics fabrication techniques and by increasing adoption of photonics components by the telecommunications industry. Tools originally developed for the telecommunications industry are now being exploited to develop novel sensors for a wide variety of applications and deployment scenarios^1–9^. Photonic sensors and communication systems are particularly valuable for operation in harsh environments, e.g. outer space and nuclear power plants, due to their small size, low power consumption, and tolerance to environmental variables such as mechanical vibrations^10–13^.

Such devices are nevertheless quite sensitive to external stimuli that produce changes in refractive index of the host material. In suitably designed photonic devices, small changes in temperature can produce measurable changes in resonance peak wavelength, thus making them useful for photonic thermometry and similar applications^9^. This sensitivity to refractive index is geometrically increased in resonant devices, like the ring resonator or photonic crystal cavity, where refractive index sensitivity grows with the device’s quality factor. Whether this sensitivity affects their usefulness in high radiation environments, however, is an open question^10,13^. Radiation induced damage is known to cause dislocations and other defects in crystalline structure that affect refractive index^14^. We recently demonstrated that Ge-doped fiber Bragg gratings (FBG) show complex dose-dependent changes in resonance peak center resulting in significant offset errors of up to ± 16.5 °C in thermometry applications^15^. These changes in Bragg resonance are independent of the polymer coating^15^ and likely derive from significant change in Ge coordination^14^.

In silicon-on-insulator (SOI) based electronic devices, trapped charges and local changes in bond structure are a known cause of device failure^16^. In principle, changes in the refractive index due to changes in free carrier population and damage to the Si lattice can significantly degrade the measurement sensitivity and accuracy of a photonic sensor. Bhandaru *et al*.^17^ have reported that unpassivated Si ring resonator devices exposed to relatively low levels of ionizing radiation (<9 kGy) show a blue shift in resonance wavelength which was not observed for passivated devices exposed to ≈1.5 kGy of ionizing radiation. The authors attributed the observed shift in unpassivated devices to accelerated growth of native oxide. Similar results have been reported for amorphous silicon and silicon nitride devices^11,12^. We note that a photonic sensor operating in a high radioactivity environment such as a nuclear power plant is expected to receive about 1 MGy^18^ of dose per year. Under such conditions it is possible that sensor performance may be negatively impacted by changes in refractive index, covalent bond breaking, radiation induced densification and/or changes in surface chemistry. Such changes would result in increased propagation losses resulting in lower quality factors and drift in resonance wavelength of resonant devices.

In this study, we systematically examine the impact of γ-radiation, up to a cumulative dose of 1 MGy (1 Gy = 100 Rad) from ^60^Co γ-ray radiation on silicon ring resonators and Bragg waveguides across multiple devices and chips. The dose absorbed by such chips was modelled using a radiation transport Monte Carlo simulation based on engineering drawings of the source and previous measurements of the radiation field. Further details of the Monte Carlo simulations used to calculate absorbed dose and the experimental setup are given in the Methods section. Our results indicate that silicon photonic devices can withstand high cumulative doses without any significant degradation in performance.

## Results

### Bragg waveguides

**Figure 1:**
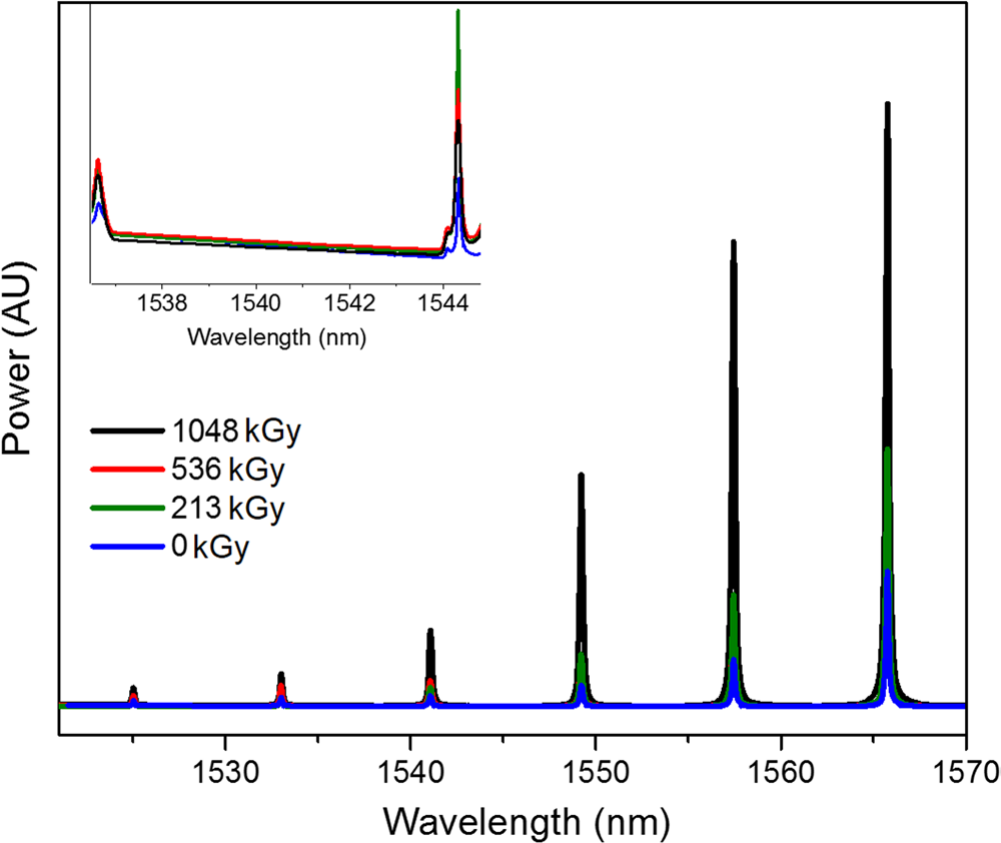
Si ring resonator and Bragg waveguide (insert) show no significant changes in spectral characteristics as absorbed dose is increased from 0 Gy to 1048 kGy (see text for details).

Bragg waveguides and ring resonators exposed to varying levels of γ-ray radiation do not show any significant changes in spectral characteristics. A typical Bragg waveguide transmission spectrum, shown in Fig. 1, does not show any systematic changes in either the peak center or the bandwidth of the Bragg waveguide rejection window. The variation in peak center observed between the different dose spectra is found to be 8 pm and is not correlated with dose (*R*^2^ = 0.28). Similarly, variation in linewidth (2.6 pm) is poorly correlated with dose (*R*^2^ = 0.54) and is within the measurement uncertainty of ±7 pm. The linewidth in a Bragg device is directly proportional to the refractive index contrast between the waveguide and etched step regions (where the evanescent field interacts with the surrounding oxide material, sampling an effectively lower refractive index than the unetched waveguide region)^19^. A lack of significant change in Bragg linewidth, therefore, suggests the oxide layer immediately next to the Si does not suffer any significant changes such as bond breaking or densification of the oxide layer due to radiation exposure. Similarly, a lack of significant change in peak isolation suggests the devices escaped with little to no damage to the Si surface or lattice.

### Ring resonators

**Figure 2:**
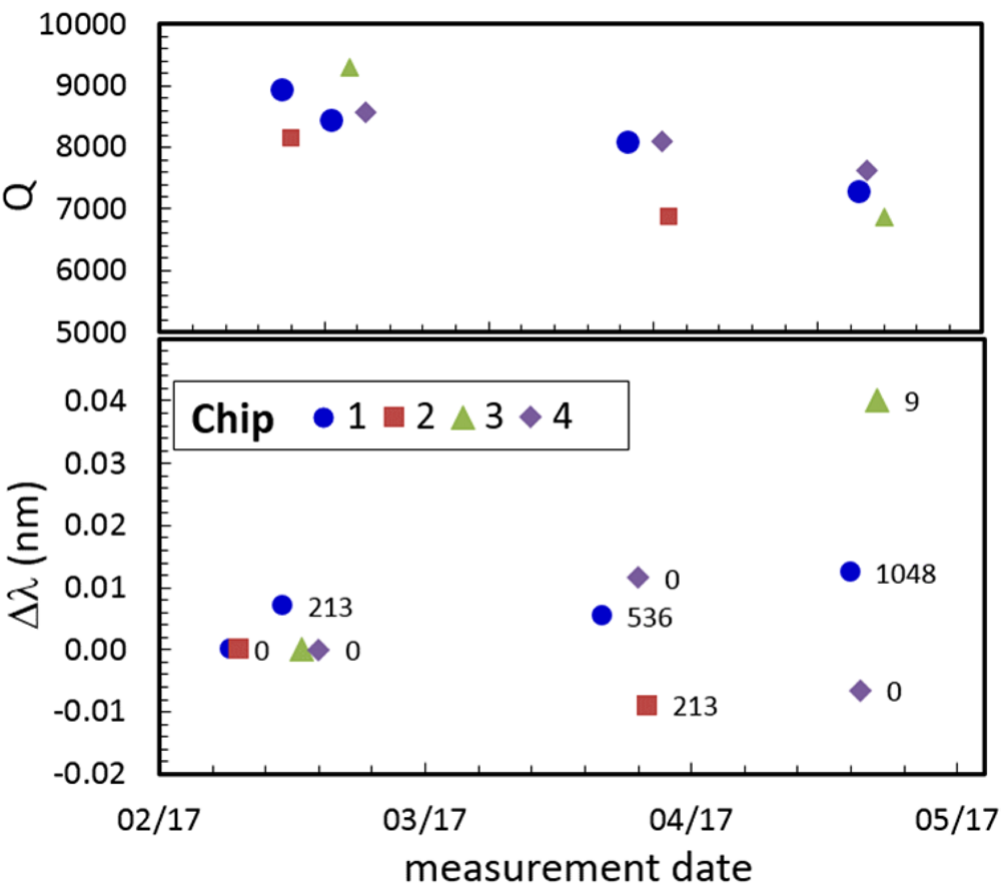
Q-value (top) and peak position (bottom) of a ring resonance across three different irradiated chips (plus control chip #4) are not significantly impacted by radiation dose (see text for discussion). Number next to the symbols refer to dose (kGy) delivered on that particular date.

**Figure 3:**
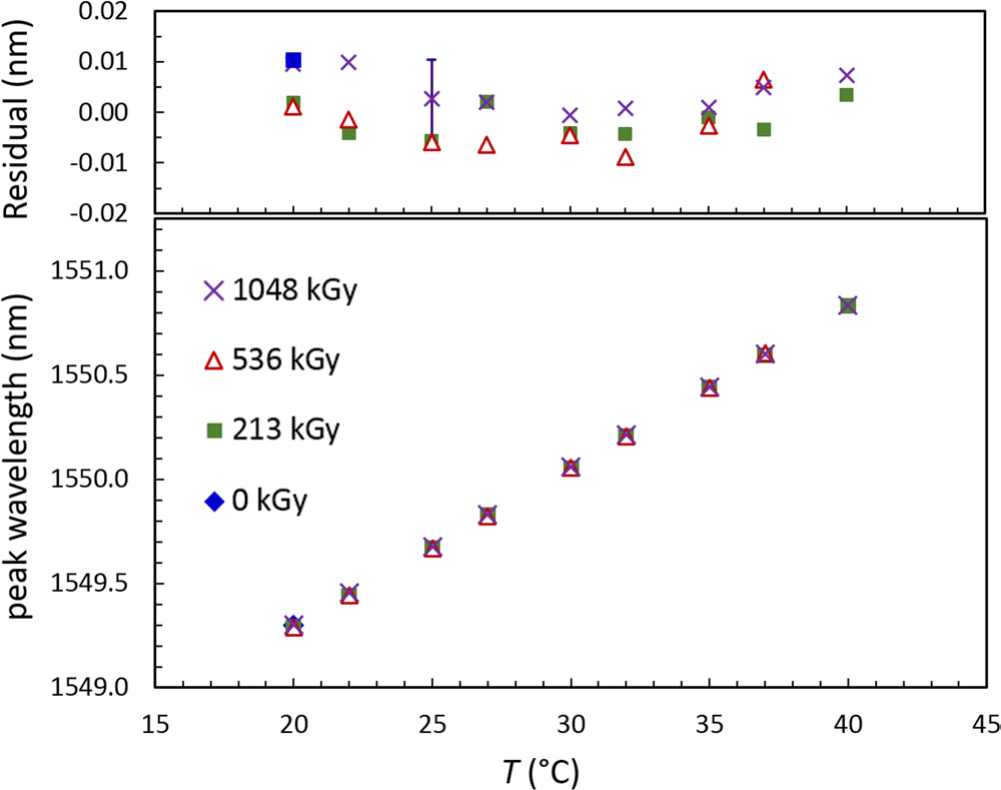
Temperature response of silicon ring resonator. Top plot shows residuals from a common fit to all the data. Propagated uncertainty due to temperature measurement is shown for one point. The temperature response is not impacted by absorbed radiation dose (see text for details).

In ring resonators, where spectral consequences of small changes in device characteristics, such as refractive index, are expected to magnify due to recirculation of light in the ring structure, we do not observe any significant dependence on absorbed dose. We have examined the impact of radiation in several ring resonator devices with quality factors (Q-factors) ranging from 5,000 to 30,000 at room temperature. As shown in Figs 2 and 3, ring resonator devices do not show any significant dose-dependent change in the free spectral range (FSR), Q-factor, and peak position. The FSR was uncorrelated with dose (*R*^2^ =0.08), with a standard deviation for the 4 doses of 2.5 pm (0.031%). The Q-factor did decrease over time, but that decrease was the same for the control chip as for the irradiated chips and thus cannot be unambiguously ascribed to dose (Fig. 2 top). The observed decrease of ≈12% in Q-factor is correlated with peak input power and is reproduced when input power is doubled, indicating the observed effect is due to the device undergoing self-heating during the laser scan, not radiation damage. The small variation in absolute peak position observed for Chips 1–3 between irradiations (average standard deviation of [13 ± 13] pm) is statistically indistinguishable to changes observed in the control chip. This small variability was found to be random, with the most stringent test coming from the peak position at 20 °C (measured at 0 Gy dose), which was found to be uncorrelated to dose, with *R*^2^ = 0.01. We note that all four chips are from the same batch and contain the same devices. The control chip traveled with the other chips to and from the photonics lab to the radiation facility but was never exposed to radiation itself.

As quantified above, the FSR of the devices does not show any significant changes, clearly indicating that neither the group index nor the dispersion (parameters important in communication systems) is impacted by radiation exposure. Examination of the temperature-dependent response of ring resonator devices shows that the temperature sensitivity is also not impacted by radiation dose. For the data shown in Fig. 3, the average and standard deviation of the three responses was (76.9 ± 0.2) pm/°C. A linear regression of response as a function of dose returned a slope of (−4 ± 2) ${10}^{-4}\frac{{\rm{pm}}}{^\circ {\rm{C}}\cdot {\rm{Gy}}}$, which was not significant at the 95% level (*t* = −1.8, *p* = 0.32). The slight offset variability observed between doses (residuals shown in Fig. 3 insert) is within the limited precision of the thin film resistance thermometer (±0.1 °C) when employed using nominal coefficients.

## Discussion

In this study, we have examined the impact of ionizing radiation on silicon photonics devices. Devices were irradiated within a self-contained, commercially produced ^60^Co irradiator, and delivered doses were estimated using well-established widely available Monte Carlo codes. Measurement of device characteristics such as peak center, peak width, FSR and temperature sensitivity do not reveal any significant dose-dependent changes, indicating that for all devices tested, the characteristic group index, dispersion and thermo-optic coefficient remain constant for aggregate doses up to 1 MGy (the maximum absorbed dose delivered in this study). These results are in stark contrast with those of FBG-based sensors, where radiation induced changes in Bragg resonance result in significant drift in device characteristics^10,13,15^. Our results bode well for efforts to leverage existing infrastructure in silicon photonics to develop communication and sensor platforms for operating in harsh environments, such as industrial sterilization of health care products or radiation processing systems where doses can be in the range 15 kGy to 300 kGy^20,21^, or nuclear power plants where dose rates of 10 kGy/h are possible^18^. Precision photonic sensors could find additional use in instrumentation used in radiotherapy clinics or space-based systems, where much lower aggregate doses (<100 Gy) are more commonplace but high reliability and accuracy are paramount^22–24^.

## Methods

### Photonic interrogation

The experimental measurement apparatus has been described in detail elsewhere^9,25^. Briefly, a C-band laser (New Focus TLB-6700 series1) is swept over the sensor resonance. Ten percent of laser power was immediately picked up from the laser output for wavelength monitoring (HighFinesse WS/7) while the rest is evanescently coupled to the photonic device under test using an optical fiber held within a few microns of the chip surface. The photonic chip itself was mounted on a Peltier assembly atop a 3-axis stage (Newport). Input from a platinum resistance thermometer (measurement accuracy ±0.1 °C) is fed to a proportional-integral-derivative controller that drives a thermoelectric cooler and maintains the temperature to within 0.02 °C of the set value. Photonic chips were fabricated at the CEA-LETI (Laboratoire d’Electronique des Technologies de l’Information, France) fabrication facility using Complementary Metal–Oxide–Semiconductor (CMOS) technology. Three of the representative chips from the batch were systematically exposed to varying levels of γ-ray radiation at the NIST high-dose dosimetry laboratory, while a fourth chip, used as a control, was never exposed to radiation, though it traveled with the other three chips between the photonics testing facility and the radiation facility.

### Gamma-ray irradiation

Photonic sensors were irradiated with γ-rays in the NIST high-dose dosimetry laboratory. Three Gammacell (GC) 220 ^60^Co irradiators (Nordion, Canada) were used with dose rates between 0.2 kGy/h and 3.9 kGy/h. Most of the irradiations were done using irradiator number GC207, which contained a nominal activity of 1.76*10^14^ Bq on a reference date of December 31, 2016 and delivered an absorbed dose rate to water, determined using alanine dosimetry, of R = 1.08 Gy/s on the reference date, with an expanded uncertainty^26^ of about 2%. This amounts to a dose rate per ^60^Co activity of *D =* 6.12*10^−15^ Gy/s/Bq.

### Monte carlo calculations of absorbed dose

Monte Carlo simulations were undertaken to calculate the dose to the silicon devices based on measured dose to alanine calibration pellets. A simplified geometry for the GC 220 was created based on the irradiator specification sheet^27^ using the EGSnrc code DOSRZnrc^28^. The 1 cm diameter ^60^Co rods were simulated as a single cylindrical shell. Following Rodrigues *et al*.^29^, aluminum and steel shells of 2 mm thickness each were implemented between the sources and the exposure chamber. The ^60^Co emission spectrum was simplified to consist of two 1.25 MeV γ-rays.

**Figure 4:**
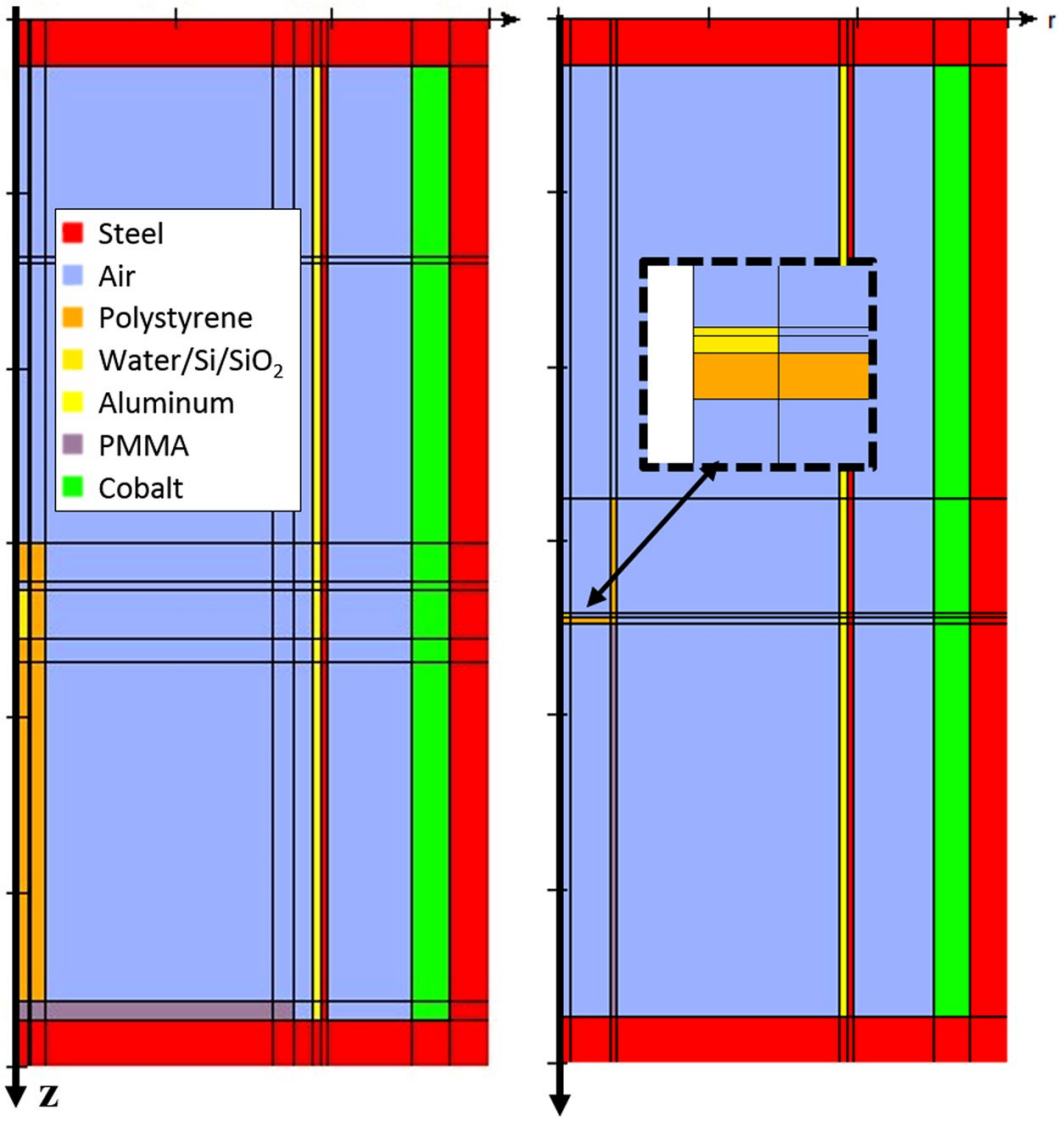
Cross-sectional side-view of the simulated geometry for the calibration pedestal (left), and chip in beaker geometry (right). The radiation absorber material in the model could be varied among water, silicon, and silica, as needed.

Two irradiation geometries were simulated- the chamber calibration and chip irradiation, shown in Fig. 4. The calibration geometry, used to transfer calibration from the primary standard of absorbed dose to water, consists of 5-mm diameter alanine pellets stacked inside a polystyrene cylinder (pedestal) of wall thickness 3.7 mm^30^. For the chip irradiation, one chip at a time was placed inside a glass beaker of diameter and wall thickness of 30.5 mm and 1.3 mm, respectively. Although the chip device layer was only a few *μ*m thick, the simulated dose to the chip was averaged over the top 100 *μ*m of the chip, to achieve adequate Monte Carlo statistics.

For the calibration geometry, the EGSnrc simulated result was *D* = 6.23 10^−15^ Gy/s/Bq, which is 2% higher than the nominal calibrated value. This agreement is adequate, considering the approximate source geometry of the EGSnrc model and the Monte Carlo counting statistics of about 2%. Despite the adequate agreement in absolute dose per activity, the EGSnrc model was not used for an absolute calculation of dose, but only used to scale the dose from the calibration geometry to the chip geometry. For the chip geometry, the EGSnrc result was *D* = 5.42 10^−15^ Gy/s/Bq. Thus, the ratio of the calculated dose for the chip geometry to the calibration geometry was 0.870, with a total Monte Carlo uncertainty of about 3%.

Scaling the calibrated value for *R* by the ratio of the EGSnrc-calculated *D* values for the chip geometry to the calibration geometry, results in a dose rate to the chip of 0.94 Gy/s with an expanded uncertainty of 7% (k = 2).

**Figure 5:**
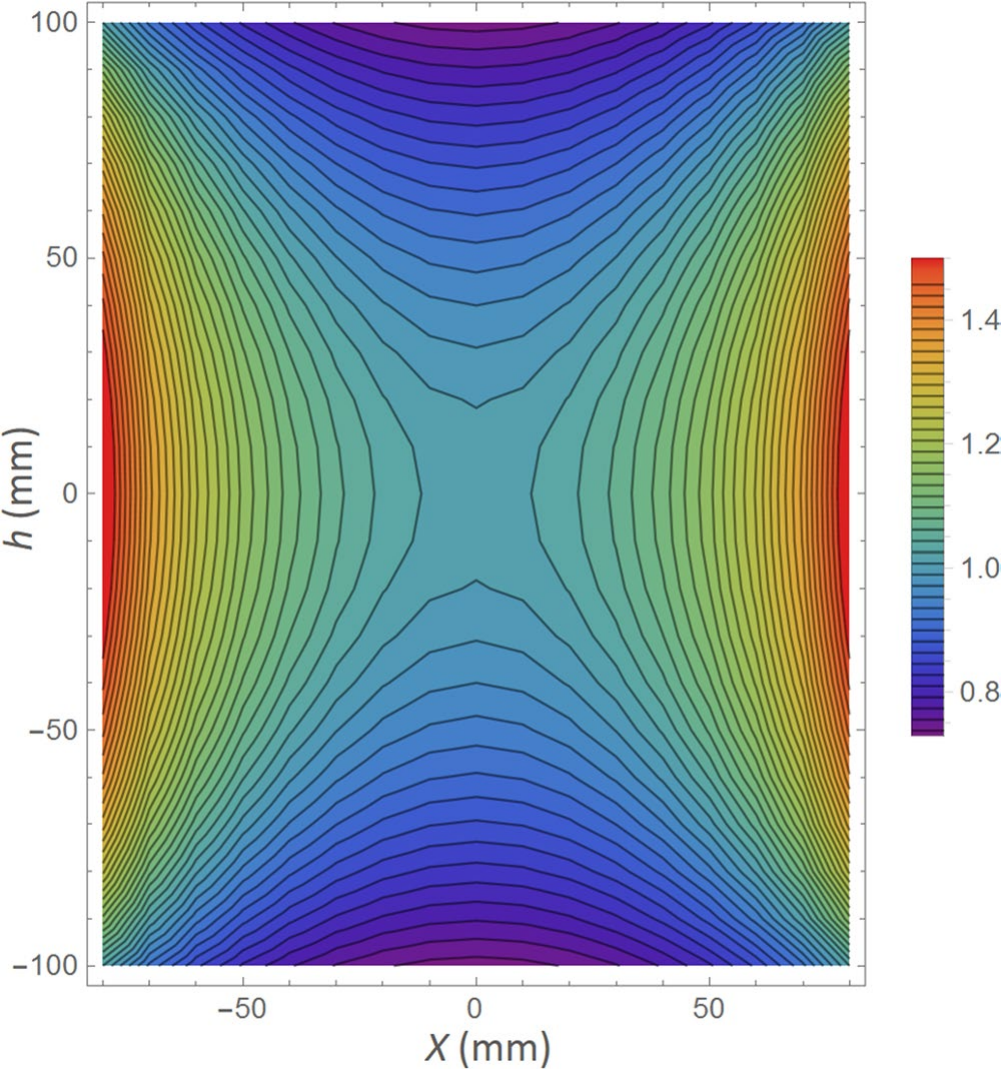
Normalized, simulated gamma-ray field, F(*x*,*h*)/F(0,0), for cylindrical shell source. Contours represent a change in field of about 2% of the central value.

An approximate gamma-ray field map was calculated by numerically solving an integral representing the *γ*-ray flux, *F*, inside a chamber consisting of a thin, radiating, cylindrical shell,

1 $$F(x,h)={\int }_{-(\frac{L}{2}+h)}^{L/2-h}{\int }_{0}^{2\pi }\frac{r}{{(r\cos (\theta )-x)}^{2}+{(r\sin (\theta ))}^{2}+{z}^{2}}d\theta dz$$

where *L* = 210 mm is the shell length, *r* = 105 mm is the shell radius, and (*x*,*h*) is the test position within the chamber. No interactions were considered. The field map, relative to the value in the center of the chamber, is shown in Fig. 5. Along the vertical axis of the chamber (*x* = 0), the field changes by −1% at *z* = ± 18 mm. Along the midplane of the chamber (*h* = 0), the field changes by +1% at *x* = ±1 mm. Therefore, the $\approx 5$ mm positioning accuracy of the chip would not significantly affect the absorbed dose beyond the Monte Carlo and calibration accuracy.

To explore the issue of transient charged particle equilibrium (TCPE), a Geant4^31^ Monte Carlo model was constructed for a version of the chip geometry. A 1.25 MeV *γ*-ray source was incident from the side of the chip, aligned with the center of the top layer, which was 200 *μ*m thick – much thicker than the actual device layer, but still not thick enough to achieve TCPE. Only about 1 out of 50 *γ*-rays interact with the chip. Of those that do, about 80% produce Compton-scattered electrons that escape into the air from the top layer. Since there is not an equal energy flux of electrons produced in the air that pass into the top Si layer, TCPE is not achieved. Therefore, the absorbed dose to the chip cannot simply be calculated based only on the relative linear-energy transfer and density of silicon relative to water. Rather, full Monte Carlo simulations are required, as were done here.

### Disclaimer

Certain equipment or materials are identified in this paper in order to specify the experimental procedure adequately. Such identification is not intended to imply endorsement by the National Institute of Standards and Technology, nor is it intended to imply that the materials or equipment identified are necessarily the best available.

## Data Availability

The datasets generated during and/or analyzed during the current study are available from the corresponding author on reasonable request.
